# Saccade suppression of displacements, but not of contrast, depends on context

**DOI:** 10.1167/jov.22.10.10

**Published:** 2022-09-09

**Authors:** Eckart Zimmermann, Joachim Lange

**Affiliations:** 1 Institute for Experimental Psychology, Heinrich Heine University Düsseldorf, Düsseldorf, Germany; 2 Institute of Clinical Neuroscience and Medical Psychology, Medical Faculty, Heinrich Heine University Düsseldorf, Düsseldorf, Germany

**Keywords:** saccade suppression, displacement detection, visual contrast

## Abstract

Saccades let the visual scene sweep with high speed across the retina, thus producing a massive motion stimulus. Yet, in natural vision, we never perceive motion that is produced by saccades. The absence of perisaccadic motion perception might be caused by a transient reduction of visual sensitivity at the time of saccade initiation, so-called saccadic suppression. Saccade suppression occurs for contrast, displacement, and motion stimuli. Saccade suppression of displacements has been shown to be context sensitive. After performing saccades in sessions without perisaccadic stimulation, saccade suppression magnitude is drastically decreased (Zimmermann, 2020). Here, we aimed to test whether saccade suppression of contrast is similarly modulated by context. To this end, we projected stimuli on a homogeneously white wall such that we could establish a ganzfeld-like environment that, depending on the experimental session, did or did not contain any visible contrast stimuli. We first successfully replicated the context sensitivity of saccade suppression of displacements. Then, we tested context sensitivity of contrast suppression by asking subjects to perform several saccades either across the uniform white wall or across a background consisting of a sinusoidal grating. In contrast to perisaccadic context sensitivity for displacement suppression, we did not find context sensitivity for suppression of contrast.

## Introduction

Every time we perform a saccade, the visual scene sweeps across the retina with high speed. Given that we perform saccades at about a frequency of 3 Hz, we should perceive saccade-induced motion every 300 ms. Yet, our perceptual experience remains undisturbed from perisaccadic motion. In general, three types of explanation have been offered to account for the lack of seeing retinal motion. The theory of saccadic active suppression claims that processing at any early visual level is transiently shut down (Burr, Morrone, & Ross, 1994). Masking theories suggest that the post-saccadic image induces backward masking of the perisaccadic sensation (Castet & Masson, 2000; Castet, Jeanjean, & Masson, 2002). The theory of the stable world assumption (Deubel, Schneider, & Bridgeman, 1996) proposes that the visual system supposes the world remain stable during saccade execution unless significant contradictory evidence is provided.

The first type of theory proposes that a dedicated mechanism transiently cancels out perisaccadic perception (Volkmann, 1986; Burr, Morrone, & Ross, 1994; Binda & Morrone, 2018). In this view, an efference copy (i.e. a copy of the motor command), informs that a saccade is upcoming. When the signal arrives in visual areas, a transient shut down around the time of saccade onset prevents sensory information to be further processed. The idea of active saccade suppression is supported by a perisaccadic reduction in sensitivity to contrast (Volkmann, 1986), displacements (Bridgeman, Hendry, & Stark, 1975), and motion (Burr, Holt, Johntone, & Ross, 1982; Shioiri & Cavanagh, 1989; Ilg & Hoffmann, 1993). Furthermore, the perisaccadic reduction in contrast sensitivity is selective for the spatial frequency of the stimulation (Burr, Morrone, & Ross, 1994). Suppression acts mostly on stimuli with low spatial frequencies, which – unlike high spatial frequencies – would result in motion experiences at saccadic speeds (Burr, Johnstone, & Ross, 1982).

The second type of theory suggests that no active mechanism is necessary to explain the absence of perisaccadic perception. Once a saccade is finished and the eye is resting again, a stationary scene will be perceived. This postsaccade sensation might mask processing of the perisaccadic stimulation (Castet, Jeanjean, & Masson, 2002). When observers perform saccades in the dark, such that no postsaccadic stimulation ensues and the light is turned on only during the saccade, they report having seen a moving image (Campbell & Wurtz, 1978). In another attempt to demonstrate that perisaccadic vision is not cancelled out when no postsaccadic stimulation is provided, researchers used a low-contrast grating that drifted too fast to be perceived with a stationary eye (Castet & Masson, 2000). As soon as a saccade was performed in the direction of the grating motion, the speed of the grating on the retina was reduced and becomes visible during the saccade. However, subjects were no longer able to discriminate the perisaccadic motion direction when postsaccadic visual stimulation was provided (Castet, Jeanjean, & Masson, 2002).

The theory of a stable world suggests that across saccade execution the visual system relies on the postsaccadic target as a reference object and assumes the world remained stable (Deubel et al., 1996). In this view, the displacement of the saccade target on the retina is detected by the M-pathway but this signal becomes suppressed by the postsaccadic image processed in the P-pathway (Takano, Matsumiya, Tseng, Kuriki, Deubel, & Shioiri, 2020). For this reason, participants were very poor in detecting displacements of the saccade target that were applied by the experimenter (Deubel et al., 1996). However, when the postsaccadic target is blanked for about 250 ms, displacement detection is almost perfect. This blanking effect demonstrates that an accurate, trans-saccadic internal target representation is available but that it becomes masked as soon as a postsaccadic target is perceived after saccade execution. The strength of the suppression, measured as displacement detection performance, depends on the contrast of the postsaccadic target (Matsumiya, Sato, & Shioiri, 2016; Grzeczkowski, Deubel, & Szinte, 2020; Takano et al., 2020). Suppression increases as a function of postsaccadic target contrast, providing evidence for masking of the M-signal via processing in the P-pathway.

Even information that is perceptually omitted during saccades is available for further processing (Watson & Krekelberg, 2009). Perisaccadic vision might be used to track objects that change position rapidly (Schweitzer & Rolfs, 2020). Perisaccadic motion streaks are generated by salient visual objects and can inform about motion in the scene. They have been shown to establish object correspondence across saccades and initiate gaze correction (Schweitzer & Rolfs, 2021).

A recent finding challenges all three theories. Retinal ganglion cells in isolated retinae of mice and pigs responded to saccade-like displacements (Idrees, Baumann, Franke, Münch, & Hafed, 2020). As a consequence, responses to additionally flashed visual stimuli were suppressed through visually triggered retinal-circuit mechanisms. In other words, the displacement of the visual scene, generated by each saccade, produces a visual stimulus which interacts with stimuli presented perisaccadically, like flashed gratings. Prima facie, the selectivity of saccadic suppression for low spatial frequencies, seems to be at odds with a retinal mechanism. However, further research by the same group demonstrated that this selectivity could be explained by purely visual interactions (Baumann, Idrees, Münch, & Hafed, 2021). Consistent with previous results (Diamond, Ross, & Morrone, 2000), Baumann et al. (2020) found that saccadic suppression is stronger when saccades translated a luminance stripe or edge across the retina compared with a uniform background. Suppression was stronger for darker backgrounds and negative polarity probe flashes. Furthermore, during simulated saccades, very similar dependencies of saccade suppression strength on visual features were found as for real saccades. These visual-visual interactions in saccadic suppression suggest that perisaccadic modulations of contrast sensitivity might have a purely visual origin and the role of the efference copy is to release perception from suppression (see Diamond, Ross, & Morrone, 2000 for the same observation).

One of us has previously shown that perisaccadic sensitivity to displacements depends on context (Zimmermann, 2020). When observers – before testing perisaccadic thresholds – perform many saccades across an empty background, perisaccadic sensitivity to displacements was significantly higher than when subjects performed saccades across a grating. Because efference copy signals can indicate the time and the size of an upcoming saccade, the brain could use the tight correlation between saccade amplitudes and motion speeds and predict which perisaccadic motion will occur. The availability of efference copy signals in motion areas has been confirmed (Berman, Cavanaugh, McAlonan, & Wurtz, 2017), thus allowing saccade plans in principle to silence the neural activity that processes the motion corresponding to the amplitude of the saccade to be performed. If storage of the sensorimotor contingency between saccades and the perisaccadic motion is responsible for the omission of the perisaccadic motion, re-learning of this contingency should alter suppression strength. Indeed, when subjects performed 100 saccades in the dark without experiencing any perisaccadic stimulation, the strength of saccade suppression to perisaccadic displacements declined. This account can explain why perisaccadic motion is omitted but not why contrast sensitivity is reduced. Consequently, according to this view, they would have to rely on separate mechanisms.

In the present study, we aimed to compare the influence of context (i.e. re-learning of certain saccades and the corresponding perisaccadic visual input), on displacements and on contrast stimuli. We first replicated the effect of context on perisaccadic displacement sensitivity. Second, we investigated whether a similar dependency between context and suppression could be observed for perisaccadic contrast sensitivity.

## General methods

### Participants

In experiment 1, there were 6 subjects (all women; mean age = 24.21 years, SD = 0.84) who participated, and in experiment 2, there were 6 subjects (all women; mean age = 24.46 years, SD = 0.76) who also participated in experiment 1, who took part in the experiment. All participants had normal or corrected-to-normal vision and gave their written informed consent. They were paid for their participation. The study was approved by the ethics committee of the Faculty of Mathematics and Natural Sciences of the Heinrich-Heine University Duesseldorf and in accordance with the 64th WMA Declaration of Helsinki.

### Saccade detection

Stimuli were projected onto a large white wall with the Acer Beamer H6502BD with a vertical frequency of 60 Hz. Subjects were seated 150 cm from the wall. The projection area extended 38 degrees × 22 degrees. In order to match the luminance of the wall with that of the stimulus projection, the wall was floodlit by two bright reflector lights that were positioned behind the back of the subject.

Eye movements were recorded by measuring the electrooculogram (EOG) while the subjects performed the experiments. EOG electrodes were placed at the outer canthi of each eye to measure vertical eye movements, and a reference was placed at the mastoid. The EOG was recorded with the BrainVision Recorder Quickamp 72 with a sampling frequency of 1000 Hz.

Continuous EOG data were offline epoched into trials using the Matlab toolbox Fieldtrip (Oostenveld, Fries, Maris, & Schoffelen, 2011). To detect saccades in each trial, EOGs were first bandpass filtered (1-15 Hz) using a fourth order Butterworth filter and Hilbert transformed to obtain the amplitude envelope. Next, the peaks in the amplitude envelope were determined. These peaks indicate the presence of a saccade (i.e. the local maxima of eye movement velocity). Only trials with a peak (i.e. a saccade) after target presentation were further analyzed whereas trials containing peaks before target presentation were discarded.

To determine the onset of a saccade, the raw EOG signal was first smoothed with a Gaussian filter of 10 ms window length. Starting 200 ms before the peak in the amplitude envelope (see above), the difference of the smoothed EOG signal in consecutive samples was calculated. If the sign of the difference was identical in a sequence of 30 consecutive samples (i.e. indicating eye movement in the same direction for at least 30 ms), the signal was identified as the saccade and the onset of the sequence determined the saccade onset.

All analyses were performed using a custom-made script in Matlab (The Mathworks Inc., Natick, MA, USA) and FieldTrip, an open source Matlab Toolbox.

## Methods experiment 1

**Figure 1.**
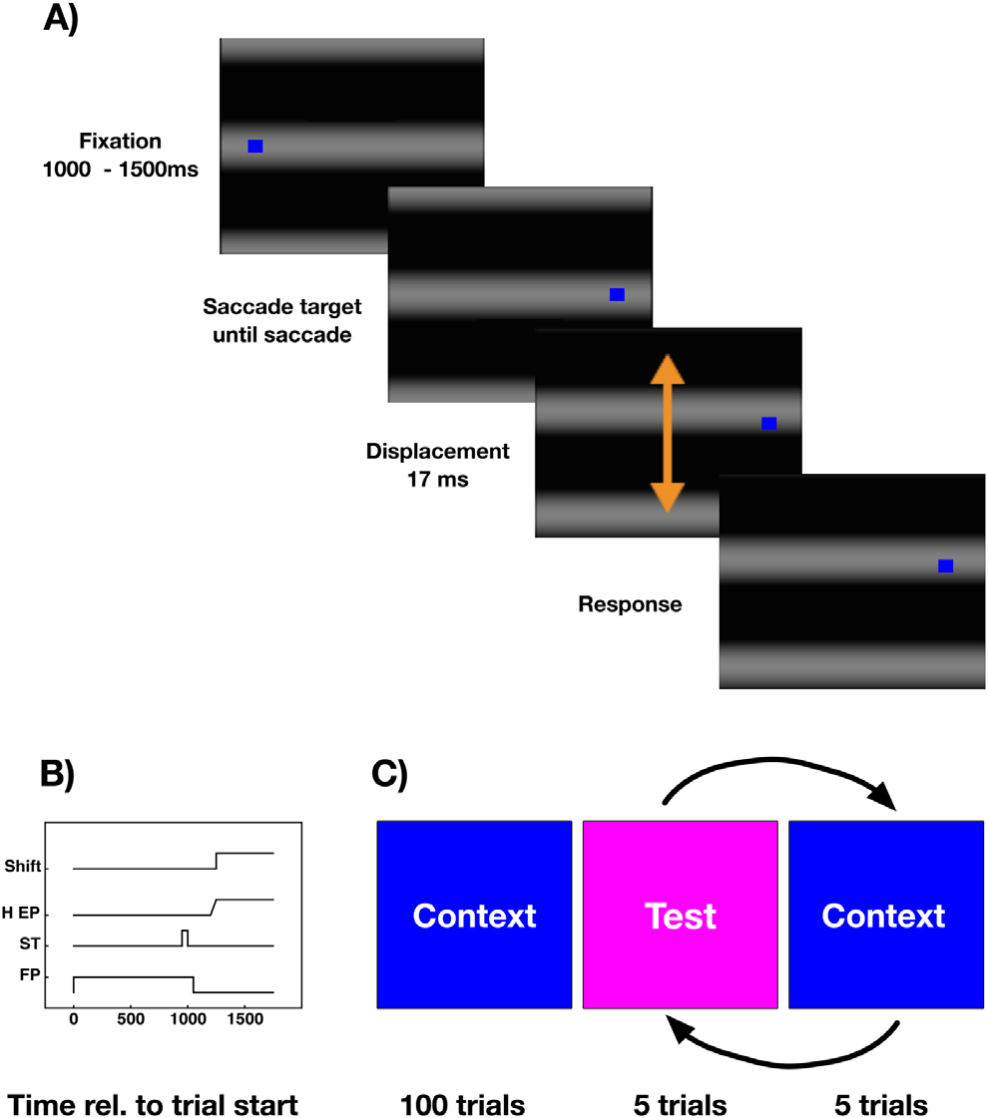
(**A**) Schematic illustration of a test trial in experiment 1. As a background, a full-field grating was presented throughout the entire experiment. A trial started with the presentation of a fixation point. After 1000 to 1500 ms, a saccade target was presented. Subjects were instructed to perform a saccade as soon as the fixation point disappeared. During saccade execution the grating was shifted either upward or downward and subjects had to estimate the displacement direction. (**B**) The time course of events in the context trials for the fixation point (FP), the saccade target (ST), the seven horizontal eye position (H EP), and the grating displacement (shift). (**C**) Graphical sketch of the trial structure for an experimental context session. Two session types were implemented. In “no context” sessions, subjects only completed test trials. In “high contrast” sessions, subjects performed 100 context trials in which the background was not displaced. After these trials, five test trials alternated with five context trials until the end of the session.

In experiment 1, two session types were tested: no context and context sessions. No context sessions contained only test trials. Figure 1A shows the structure of a test trial. A test trial started with the presentation of a fixation point, which was shown on top of a full-screen horizontal sinusoidal grating (black = lum = 77.2 cd/m2 and white = lum = 87.3 cd/m2) grating, spatial frequency = 0.14c/degrees, Michelson-contrast: 0.06). Subjects were required to direct their gaze onto the fixation point which was shown 19 degrees in the left periphery for a random period between 1000 and 1500 ms (see Figure 1B). Simultaneously with the disappearance of the fixation point, a saccade target was presented 19 degrees in the right periphery. Participants were instructed to perform a saccade to the target as soon as it appeared. In order to measure the time course of saccade suppression at various times (i.e. 150 ms–180 ms after the presentation of the saccade target), the background was displaced by 50 degrees (rotational degree) either in an upward or downward direction. A previous study had shown that with this displacement size, subjects are well able to judge the displacement direction correctly when it occurs before or after saccade onset but not around the time of saccade initiation (Zimmermann, 2020). After subjects had executed their saccades, they had to report the direction of the background displacement by using the corresponding arrow keys on the computer keyboard using their right hand. Context sessions included test trials and context trials. In context trials, the background was lit homogeneously gray. As in test trials, subject had to fixate on the fixation point and to perform a saccade to the saccade target as soon as it appeared. In context trials, the subjects had no other task than performing the saccade. Context sessions started with 100 context trials after which, in another 100 trials, five test trials alternated with five context trials until the end of the experiment (see Figure 1C). Context sessions contained 200 trials in total, baseline sessions contained 100 trials in total. At the end of a context trial, participants pressed one of the response buttons to start the next trial. It could be argued that baseline sessions should have contained context trials in which the grating was displaced. However, in a previous study (Zimmermann, 2020), it was found that providing a context with displacements or providing no context at all yields the same result. For this reason, we chose no-context trials in the present study. On average, participants completed 665.17 (SEM = 80) trials.

### Data analysis

To analyze performance as a function of time relative to saccade onset, we binned the responses into bins with a width of 35 ms. To quantify baseline contrast sensitivity, we averaged across all data within each subject and each condition for which stimuli were presented between 200 ms and 75 ms before saccade onset and data for which stimuli were presented between 75 ms and 200 ms after saccade onset. To quantify perisaccadic sensitivity, we chose the bins that fell into the perisaccadic range (−25 ms −10 ms relative to saccade onset). Within each bin we averaged across responses to obtain the proportion correct. For statical analyses in both experiments, we calculated a nonparametric ANOVA, using the Aligned Rank Transform (Wobbrock et al., 2011). In addition, Bayes factors were calculated for the comparison of perisaccadic sensitivity in context and no context (or no contrast in experiment 2) sessions using the statistics software JASP.

## Results experiment 1

**Figure 2.**
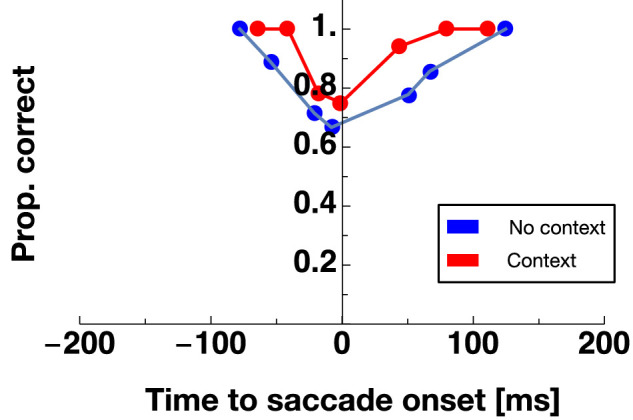
Proportion of correct displacement discrimination as a function of stimulus presentation time relative to saccade onset for one example observer. Data from no context sessions is shown in blue and data from context sessions in red. Responses are binned into bins with a width of 35 ms.

**Figure 3.**
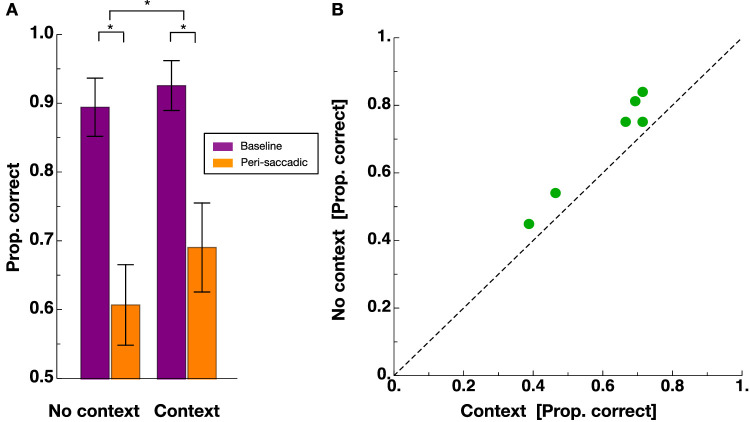
(**A**) Average proportion of correct displacement discrimination averaged across all observers. Data from the baseline range are shown in purple and data falling into the bin of the peri-saccadic range are shown in orange. Error bars represent SEM. (**B**) Average perisaccadic displacement discrimination from no context sessions against those from context sessions for all observers.

In experiment 1, participants had to perform a saccade to a target. At various times around saccade initiation, the background – consisting of a grating – was displaced either upward or downward and the subjects had to report the direction of the displacement. Figure 2 shows proportion correct of displacement discrimination as a function of the time relative to saccade onset for an example participant. Data from no context sessions are shown in blue and data from context sessions in red. One can see that discrimination performance is close to one (i.e. optimal discrimination performance), long before (<−100 ms) or long after (>100 ms) saccade onset. However, performance declines the closer in time displacements were presented to saccade onset. This result replicates previous findings (Bridgeman, Hendry, & Stark, 1975; Zimmermann, 2020). One can also see that the peak of suppression at the time of saccade onset is lower in no context sessions than in context sessions. In other words, after performing context trials, subjects were better able to discriminate the direction of the background displacement. Figure 3A shows results averaged across all subjects with discrimination performance measured in the baseline range (shown in purple) and within the perisaccadic range (shown in orange). The reduction in discrimination performance in the perisaccadic range can be seen clearly for both session types. A nonparametric repeated measures ANOVA with the factors “stimulus time” (baseline/perisaccadic) and “session type” (“no context”/“context”) revealed a significant main effect for the factor “stimulus time” (F(1,5) = 18.85, *p* = 0.007), confirming the well-known suppression of displacements during saccades (Bridgeman, Hendry, & Stark, 1975). The significant main effect “session type” (F(1,10) = 24.96, *p* = 0.0005) and the significant interaction effect (F(1,10) = 5.63, *p* = 0.03) confirmed that we successfully replicated the previously reported dependency of perisaccadic displacement sensitivity on context (Zimmermann, 2020). Strong evidence in favor of the alternative hypothesis (i.e. a difference in perisaccadic displacement sensitivity between context and no context sessions) was provided by a Bayes factor of 22.97. Please note that the effect could be demonstrated for each subject (see Figure 3B).

## Methods experiment 2

**Figure 4.**
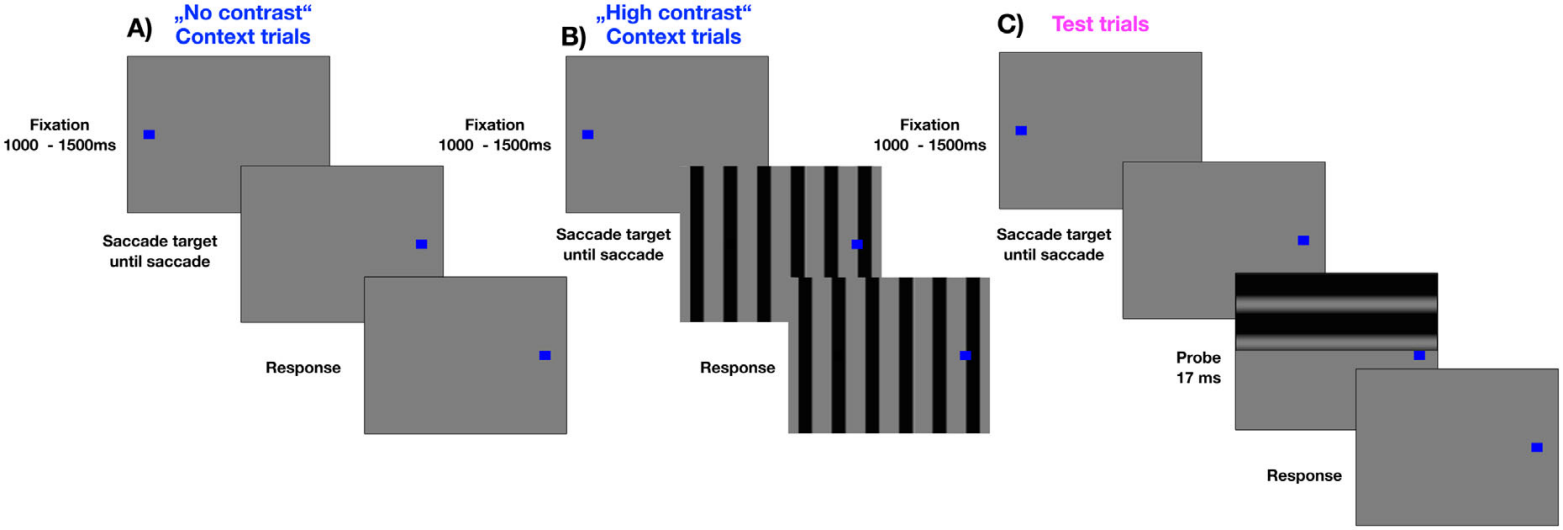
(**A**) Schematic illustration of a “no contrast” context trial in experiment 2. In the “no contrast” context sessions no background grating was presented. A trial started with the presentation of a fixation point. After 1000 to 1500 ms, a saccade target was presented. Subjects were instructed to perform a saccade as soon as the fixation point disappeared. (**B**) Schematic illustration of a “high contrast” context trial in experiment 2. In the “high contrast” context sessions, a vertical full-field grating appeared simultaneously with the saccade target. In all other respects, the trial structure was identical to “no contrast” context trials. (**C**) Schematic illustration of a test trial in experiment 2. A trial started with the presentation of a fixation point. After 1000 to 1500 ms, a saccade target was presented. Subjects were instructed to perform a saccade as soon as the fixation point disappeared. Around saccade execution, a horizontal grating was briefly flashed either in the upper or in the lower half of the visual field. The subject had to report the location where the grating was flashed.

In experiment 2, we asked whether the same dependency of suppression strength on context would be found for perisaccadic sensitivity of contrast. To this end, we created two contrast contexts. In the “no contrast” context trials (see Figure 4A), subjects performed saccades across the white wall without any additional background. In the “high contrast” context trials (see Figure 4B), a vertical grating (black = lum = 77.2 cd/m2 and white = lum = 208.2 cd/m2 grating, spatial frequency = 0.68c/degrees) appeared simultaneously with the saccade target. We presented the grating orthogonal to the saccade path to maximize the experience of the grating contrast. Because subjects performed rightward saccades, we chose a vertical grating orientation. In test trials (see Figure 4C), we flashed a horizontal grating at various times across saccade execution in the lower or upper half of the screen. The horizontal grating orientation was chosen in test trials because the bars of the grating being parallel to the saccade path would not produce motion and thus would not interfere with their detection. Subjects had to report the location of the grating (up or down) on the computer keyboard. Contrast (0.01–0.061 Michelson contrast) and spatial frequency (0.11, 0.17, 0.34, and 0.68 c/degrees) of the flashed probe gratings were varied across trials. On average, participants completed 670 (SEM = 99.8) trials.

### Data analysis

We estimated subjects’ contrast thresholds by plotting proportion correct against stimulus contrast and by fitting the distribution with a cumulative gaussian function (varying between 50% and 100% correct); the median of the fit estimated the visibility threshold and sensitivity was computed as the inverse of this value. In all analyzes for experiment 2, we have aggregated data across spatial frequencies.

## Results experiment 2

**Figure 5.**
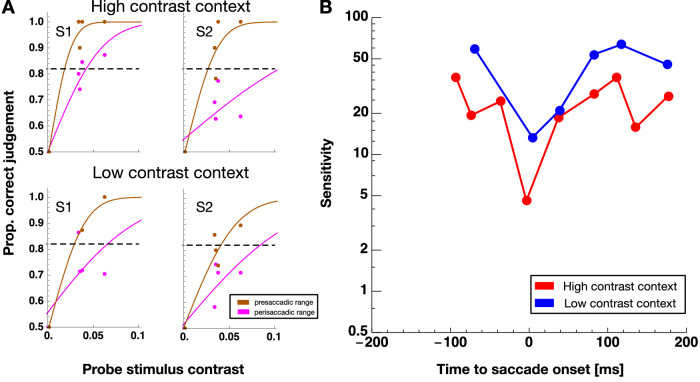
(**A**) Example psychometric functions of two subjects showing proportion of correct grating location reports against probe stimulus contrast. Data from the presaccadic range are shown in brown and data from the perisaccadic range in magenta. (**B**) Proportion of correct grating location reports as a function of stimulus presentation time relative to saccade onset for one example observer. Data from “low contrast context” sessions is shown in blue and data from “high contrast context” sessions in red.

**Figure 6.**
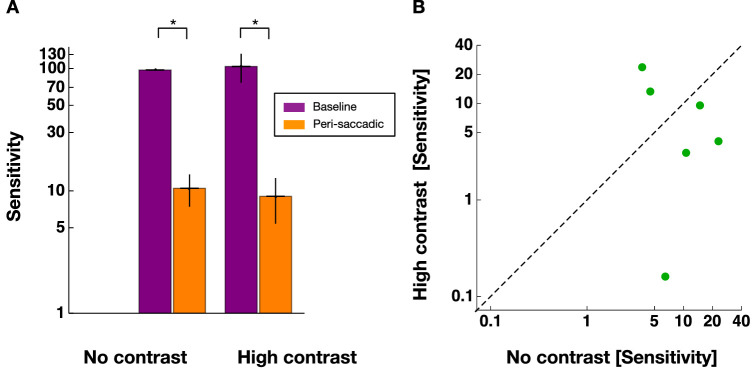
(**A**) Contrast thresholds averaged across all observers for both contexts. Data from the baseline range are shown in purple and data from the peri-saccadic range in orange. Error bars represent SEM. (**B**) Average perisaccadic sensitivity from “no contrast” sessions against those from “high contrast” sessions for all observers.

Figure 5A shows psychometric functions measured in the pre and the perisaccadic ranges. One can see that performance, indicated by the slope of the psychometric function is higher in the pre than the perisaccadic range. The time course of contrast sensitivity as a function of stimulus presentation time relative to saccade onset is shown in Figure 5B for one example subject. For both, the “no contrast” (shown in blue) and the “high contrast” (shown in red) context sessions the classical saccade contrast suppression can be seen where sensitivity is high before and after the saccade and declines around saccade onset. For the subject shown, saccade suppression is stronger in the high contrast sessions. Figure 6 shows contrast thresholds averaged across all subjects. One can see a clear difference in baseline and perisaccadic thresholds for both contrast context condition. A nonparametric ANOVA with the factors “session type” (high contrast / no contrast) and “stimulus time” (baseline/perisaccadic) revealed a significant main effect for the factor “stimulus time” (F(1,5) = 30,857, *p* < 0.003), confirming that contrast sensitivity was suppressed at the time of saccade onset. The factor “session type” (F(1,10) = 0.672, *p* = 0.431) and the interaction (F(1,10) = 1.470, *p* = 0.253) were not significant. Under the hypothesis that a contrast context would increase perisaccadic sensitivity compared with the no-contrast context, we found evidence in favor of the null hypothesis with a Bayes factor of 1 of 3.18.

## Discussion

In this study, we show that the magnitude of saccadic suppression for displacements but not for contrast is modulated by context. With the demonstration of context sensitivity for displacements we confirm previous results (Zimmermann, 2020). We add to these findings that perisaccadic suppression of contrast is not determined by the recent saccades’ context. Performing a hundred saccades across a homogeneously white wall had the same effect on the magnitude of saccade contrast suppression as the execution of the same amounts of saccades across a background containing a sinusoidal grating.

Early research on saccadic suppression focused mostly on the perisaccadic elevation of contrast thresholds (for a review, see Binda & Morrone, 2018). The specific perisaccadic contrast suppression of low-spatial frequency stimuli and the absence of suppression for high spatial frequency and color stimuli has been interpreted as evidence that at the time of saccade execution, information processing in the M-pathway is transiently suspended (Burr, Morrone, & Ross, 1994). The perisaccadic shutdown of neural processing at an early visual level would also explain why we do not perceive the motion that is produced on the retina whenever we perform a saccade, a phenomenon termed saccade omission (Watson & Krekelberg, 2009). In this theory, contrast suppression and motion omission are consequences of the same active process. More recent results suggest that color stimuli are suppressed too, although not as strong as low spatial frequency information (Braun, Schütz, & Gegenfurtner, 2017). The theory of active suppression has been challenged, however, by demonstrations of clear perisaccadic motion perception (Castet & Masson, 2000). Using stimuli that could only be detected during saccade eye movements, Castet and Masson (2000) showed that subjects were able to see external motion during saccade execution. As soon as the postsaccadic stimulation was available, motion perception vanished, indicating that backward masking might explain why in real-life viewing we do not see the motion that is produced by the saccade. In this passive theory of suppression, perisaccadic omission of motion is the consequence of backward masking by the postsaccadic image. By contrast, the theory of the stable world assumption does posit a suppression mechanism for the visual transient produced by the retinal displacement (Takano et al., 2020). They argue that creating a simulated saccade condition is impossible without inducing the perception of a visual transient. The perisaccadic visual transient, in their view, is masked by the processing of the postsaccadic image. We argue for an additional role of motion processing in the suppression of displacements. In our displacement task, the background was shifted in the vertical direction for one frame. Detection of the displacement could be accomplished by comparing the pre and the postsaccadic image or by perception of the motion transient or by both. Because visual motion for perisaccadic motion is reduced (Burr et al., 1982; Shioiri & Cavanagh, 1989; Ilg & Hoffmann, 1993), the perisaccadic suppression of displacement detection is to be affected by motion processing in addition to masking by the postsaccadic image.

In the design of the context conditions, we did not intend to induce perceptual adaptation. Indeed, in the previous study (Zimmermann, 2020), we did not observe adaptation aftereffects that might have been induced by the context trials, because biases in displacement discrimination remained unchanged by the context. For the contrast context in the present study, we similarly avoided to induce perceptual adaptation. The context, consisting of a grating, was presented only shortly around saccade execution and had an orientation orthogonal to the probe grating. We aimed to test the idea of a general modulation of contrast sensitivity by context. Perisaccadic contextual modulation that is feature-specific, like contrast adaptation, would hardly be a plausible mechanism for saccadic suppression in real life environments. Gu et al. (2014) have investigated the effect of contrast adaptation on saccade suppression of contrast. They found that saccade suppression decreased as a function of luminance contrast used during adaptation.

If the same context trials release displacement detection but not contrast from suppression, then suppression of contrast and omission of motion most likely occur at separate stages. In our first experiment, we presented upward displacements during each saccade in the no-context trials. In the context trials, test trials were counterbalanced by the same number of context trials (plus a hundred context trials at session start). We suggest that the different expectations about the perceptual saccade contingencies explain the differences in suppression magnitude in our experiment 1. The preparation of a saccade plan activates neurons in visual areas before the saccade has been initiated, thereby reducing the neuron's responsiveness for stimuli presented in the perisaccadic period. Such a pre-activation of visual areas by saccade plans can be arranged by an efference copy (Wurtz, 2018). Predictive effects of saccades on visual areas have been shown in electrophysiological (Nakamura & Colby, 2002), brain imaging (Vallines & Greenlee, 2006), and electroencephalogram (Kern et al., 2021) studies. The proposed mechanism would saturate neurons to the information most prevalent in the previous saccadic context and cancel out perisaccadic stimulation that is most likely to occur. This would explain why we are not actually blind in the presaccadic period as we can, for instance, perceive motion if stimuli are made optimal to compensate for the retinal displacement (Castet & Masson, 2000). From the present and past data, we can narrow down in which areas neurons become saturated around saccade execution. In a previous study (Zimmermann, 2020), one of us showed that when participants performed context trials containing vertical displacements, they had stronger suppression in test trials when the displacement direction in context and test trials matched than when it was in opposite directions. The neurons should be sensitive to the direction of displacements. These could be either motion-direction – selective cells or cells that become saturated by the repeated exposure to a certain form. From the present study, we can infer that the neurons should be located upstream of a contrast processing stage, because no effect on perisaccadic contrast perception was found. Taken all together, our data provide evidence for separate processes involved in contrast suppression and saccadic omission. This interpretation is consistent with the idea that the efference copy does not lead to the production of saccade suppression but to its quicker recovery (Diamond, Ross, & Morrone, 2000; Baumann et al., 2021).

Under the assumption that contrast suppression and motion omission are generated by separate mechanisms, it is possible that contrast suppression can be modified only after much more trials. Consistent with this assumption, a recent study reported that perceptual learning over a period of 7 days can silence saccadic suppression (Scholes, McGraw, & Roach, 2021). During training, subjects had to detect a low-contrast grating that was embedded in dynamic noise. Saccade suppression magnitude for these stimuli decreased as a function of training duration.

In conclusion, our data reveal that perisaccadic displacement suppression but not perisaccadic suppression of visual contrast is modulated by the history of intrasaccadic stimulation during the most recent saccades. These results indicate different mechanisms for perisaccadic contrast and motion perception.
